# Modulation of riboflavin biosynthesis enzymes in non-typhoidal *Salmonella* isolates differentially impacts human MAIT cell activation

**DOI:** 10.1371/journal.ppat.1014715

**Published:** 2026-11-03

**Authors:** Leia Vainman, Corinna A. Kulicke, Nurudeen Oketade, Noa Bossel Ben-Moshe, Camilla Ciolli-Mattioli, Aryeh Solomon, Jay C. D. Hinton, Karen M. Dobos, David M. Lewinsohn, Shelly Hen-Avivi, Roi Avraham

**Affiliations:** 1 Department of Immunology and Regenerative Biology, Weizmann Institute of Science, Rehovot, Israel; 2 Division of Pulmonary, Allergy, and Critical Care Medicine, Oregon Health and Science University, Portland, Oregon, United States of America; 3 Department of Microbiology, Immunology and Pathology, Colorado State University, Fort Collins, Colorado, United States of America; 4 Helmholtz Institute for RNA-based Infection Research (HIRI), Helmholtz Centre for Infection Research (HZI), Würzburg, Germany; 5 Faculty of Medicine, University of Würzburg, Würzburg, Germany; 6 Institute of Infection, Veterinary and Ecological Sciences, University of Liverpool, Liverpool, United Kingdom; 7 VA Portland Health Care System, Portland, Oregon, United States of America; University of Pennsylvania, UNITED STATES OF AMERICA

## Abstract

Invasive non-typhoidal *Salmonella* (iNTS) sequence type 313 (ST313) isolates have emerged in sub-Saharan Africa, with a high incidence of multidrug resistance, enhanced intracellular persistence, increased dissemination and immune evasion. ST313 isolate D23580 was shown to avoid activation of mucosal-associated invariant T (MAIT) cells compared with a genetically similar, non-invasive *Salmonella* Typhimurium (*S*.Tm) 4/74 strain, but the mechanism remains unclear. Here, we studied immune responses to infection with typhoidal strains and ST313 isolates using comparative transcriptomic profiling of human peripheral blood mononuclear cells (PBMCs). MAIT cells responded to typhoidal strains but not to D23580. To uncover underlying mechanisms, we engineered an inducible CRISPRi system targeting riboflavin biosynthesis enzymes (*rib*), which process MAIT ligands, in D23580 and 4/74 strains. Several *rib* hypomorphs exhibited prolonged lag time in minimal media, which was more pronounced in D23580 than in 4/74. In PBMC infection, knockdown of lumazine synthase (RibH) and riboflavin synthase (RibE) resulted in contrasting MAIT cell activation phenotypes. RibH knockdown restored MAIT cell activation in D23580 but did not affect activation in 4/74. Conversely, RibE knockdown diminished MAIT cell activation by 4/74 but had no impact on MAIT cell activation by D23580. Metabolomic analysis revealed accumulation of the biosynthetic intermediate DMRL in RibE knockdown and a differential impact on riboflavin biosynthesis end products between D23580 and 4/74 RibH knockdown, suggesting altered pathway flux between the strains. Our results indicate RibH and RibE as pivotal nodes for strain-specific MAIT-activation during *Salmonella* infection of human PBMCs, illustrating teir central role in the complex interplay between bacterial metabolism and host immunity.

## Author summary

Invasive non-typhoidal *Salmonella* (iNTS) is a major cause of bloodstream infections in sub-Saharan Africa, where it causes severe disease and high mortality. A particular lineage, sequence type 313 (ST313), has evolved the ability to evade immune responses, including activation of mucosal-associated invariant T (MAIT) cells, an important component of the early antibacterial immune response. MAIT cells recognize small molecules generated during bacterial riboflavin (vitamin B2) biosynthesis, but how ST313 avoids this recognition has remained unclear. Here, we compared immune responses to invasive and non-invasive *Salmonella* strains and used an inducible CRISPR interference system to selectively reduce expression of riboflavin biosynthesis genes. We found that two enzymes, RibH and RibE, have distinct and strain-specific effects on MAIT cell activation. Reducing RibH expression restored MAIT cell activation in the invasive ST313 strain, whereas reducing RibE impaired MAIT cell activation in the non-invasive strain. Metabolomic analyses showed that these genetic perturbations altered riboflavin pathway intermediates differently in each strain, revealing unexpected differences in metabolic flux. Our findings demonstrate that changes in bacterial vitamin B2 metabolism can profoundly influence immune recognition, providing new insight into how invasive *Salmonella* adapts to evade host immunity.

## Introduction

*Salmonella enterica* are Gram-negative, intracellular pathogens comprising over 2,600 distinct serovars. Different serovars exhibit significant variability in host specificity and disease manifestation, ranging from self-limiting gastroenteritis to invasive systemic infections [1]. *Salmonella enterica* serovar Typhi (*S.*Ty) and *Salmonella enterica* serovar Paratyphi A (*S.*Pty A) are responsible for an estimated nine million cases of typhoid and paratyphoid fever annually, leading to over 100,000 fatalities worldwide [2]. In contrast, non-typhoidal *Salmonella* (NTS) serovars, which infect a broad range of hosts, typically present as acute gastroenteritis in humans. The emergence of multidrug-resistant invasive non-typhoidal *Salmonella* (iNTS) variants, particularly in Sub-Saharan Africa, has led to a concerning increase in typhoid-like disease among children, elderly and immuno-compromised individuals [3,4]. The number of cases for iNTS was estimated at over half a million and over 60,000 fatalities in 2019 [4]. Notably, bloodstream isolates belong to *Salmonella enterica* serovar Typhimurium (*S*.Tm) [5,6], with the predominant variant *S.*Tm Sequence Type (ST) 313 exhibiting enhanced invasiveness and systemic dissemination. *S.*Tm ST313 also exhibits hyper-replication within human macrophages and neutrophils [7]. This increased pathogenic potency [8–10] has been attributed to host-restricted evolution [8,11,12].

The ability of ST313 strain variants to efficiently infiltrate the bloodstream has been previously linked to their capacity to evade immune responses. Notably, *S*.Tm strain D23580 only elicits weak activation of Mucosal-Associated Invariant T (MAIT) cells during *ex vivo* infection of human peripheral blood mononuclear cells (PBMCs) [13]. MAIT cells are a subset of innate-like effector T cells that play a role in immune responses [14,15]. MAIT cells contribute to host defense through the secretion of pro-inflammatory cytokines, recruitment of immune cells to infection sites, promotion of monocyte differentiation, and direct cytotoxic activity [16–18]. An *in vivo* human typhoid challenge model suggests that MAIT cells have a role in the immune response of patients who developed disease symptoms [19]. In humans, MAIT cells are widely distributed across mucosal tissues, blood, lungs, skin and spleen [15]. Activation of MAIT cells in response to bacterial infection occurs through the presentation of specific ligands bound to the antigen-presenting molecule MHC class I-related protein (MR1) on antigen-presenting cells (APCs) [20–22]. The best characterized MAIT cell-activating ligands are derived from the riboflavin (RF) biosynthesis pathway [16,20,23]. RF, also known as vitamin B_2_, is an essential precursor for flavin mononucleotide (FMN) and flavin adenine dinucleotide (FAD), which serve as critical cofactors in various biochemical processes across all domains of life. Bacteria that lack the ability to synthesize RF can obtain it from the environment through dedicated RF transporters [24,25]. While plants, fungi, and certain bacteria synthesize RF via the enzymatic conversion of guanosine triphosphate (GTP) and ribulose-5-phosphate (Ru-5-P) through the action of *rib* enzymes, humans and animals lack this biosynthetic capability and instead rely on dietary intake and microbial contributions from the gut microbiota [26,27]. Notably, while RF itself does not elicit an immune response, intermediates of the pathway can yield potent MAIT cell–activating ligands. In particular, 5-amino-6-(1-D-ribitylamino)uracil (5-A-RU) reacts with small carbonyl compounds, such as methylglyoxal (MG), forming unstable adducts that are bound by MR1 and recognized by the MAIT cell T-Cell Receptor [28]. Downstream intermediates such as 6,7-dimethyl-8-(D-ribityl)lumazine (DMRL) are not considered primary MAIT-activating antigens [26,28].

*Salmonella* spp. encode the enzymes necessary for RF production [27,29]. Despite harboring conserved *rib* genes, *S*.Tm ST313 lineage 2 strain D23580 exhibits a markedly reduced capacity to stimulate MAIT cells [13] compared to other *Salmonella* serovars, including *S*.Tm ST19 strain 4/74, which shares 92% of coding sequences (CDS) and 95% of small RNA (sRNA) sequences with D23580 [19,20,30]. In this work, we studied the molecular determinants in the RF pathway that underlie altered MAIT cell activation following infection with D23580 in comparison to other *Salmonella* serovars and strains. Using conditional knockdown of each *rib* enzyme, we experimentally revealed a central role for RibH and RibE as key determinants of differential MAIT cell activation during infection with different *Salmonella* strains.

## Results

### Single-cell RNA sequencing revealed serovar-specific MAIT cell activation

To study cell-type-specific human immune responses to different human-adapted *Salmonella* serovars, we infected PBMCs from a healthy donor with typhoidal serovars *S*.Ty str. Quailes, *S*.Pty A str. NVGH308, and iNTS *S*.Tm ST313 str. D23580. PBMCs were infected at a multiplicity of infection (MOI) of 1. At 30 minutes post infection, cells were washed with gentamicin to eliminate extracellular bacteria. Four hours post-infection (h.p.i) PBMCs were incubated with hash-tag antibodies [31], pooled, processed, and analyzed by single-cell RNA sequencing (scRNA-seq) (Fig 1A). After filtering, 6,758 high-quality cells were retained for downstream analysis: 1,300 naïve cells, 1,889 from *S*. Tm infection, 1,625 from *S*.Ty infection, and 1,944 from *S*.Pty A infection. Cell types were identified using known marker genes and further partitioning using a Louvain community detection algorithm classified 21 subtypes (Fig 1B and S1A-B Fig). Within monocytes (CD14/16, LYZ, FCGR2A, TNFAIP2, IRG1), classical monocytes were annotated based on expression of CD14, S100A8, and S100A9. M1-like monocytes were identified based on the expression of pro-inflammatory cytokines TNF, CCL3, CCL4, and CXCL8. Non-classical (NC) monocytes were annotated based on the expression of CD14, FCGR3A (CD16), and CD163. Two NC monocyte populations expressed higher levels of genes associated with oxidized low-density lipoprotein (OxLDL), such as LIPA, LGMN and CD36, a receptor responsible for OxLDL uptake in macrophages [32,33]. This metabolic state was previously shown to elevate intracellular levels of reactive oxygen species (ROS) [34], and provide an intracellular replication niche for *S.*Tm [35]. One of these OxLDL NC monocyte clusters additionally expressed a distinct M2-like signature, with elevated levels of MRC1, CD163, RSAD2, GLUL, CCL2, CCL8, and CCL7. Notably, these M2-like CCL7 OxLDL monocytes were predominantly observed in infection with D23580 but were very low in *S.*Ty and *S.*Pty A infections (S1B Fig). Within T cells (CD3D, CD3E, CD4, CD8A and CD8B), MAIT cells expressing CD8A, KLRB1 (CD161) and GPR171, presented a different activation status between infection conditions. While the total frequency of MAIT cells (activated and naïve) was comparable between conditions, activated MAIT cells expressing pro-inflammatory genes IFNγ, TNF, CCL20 and GZMB were detected following infection with *S.*Ty and *S.*Pty A, but were very low following D23580 infection (Fig 1C-D).

**Fig 1:**
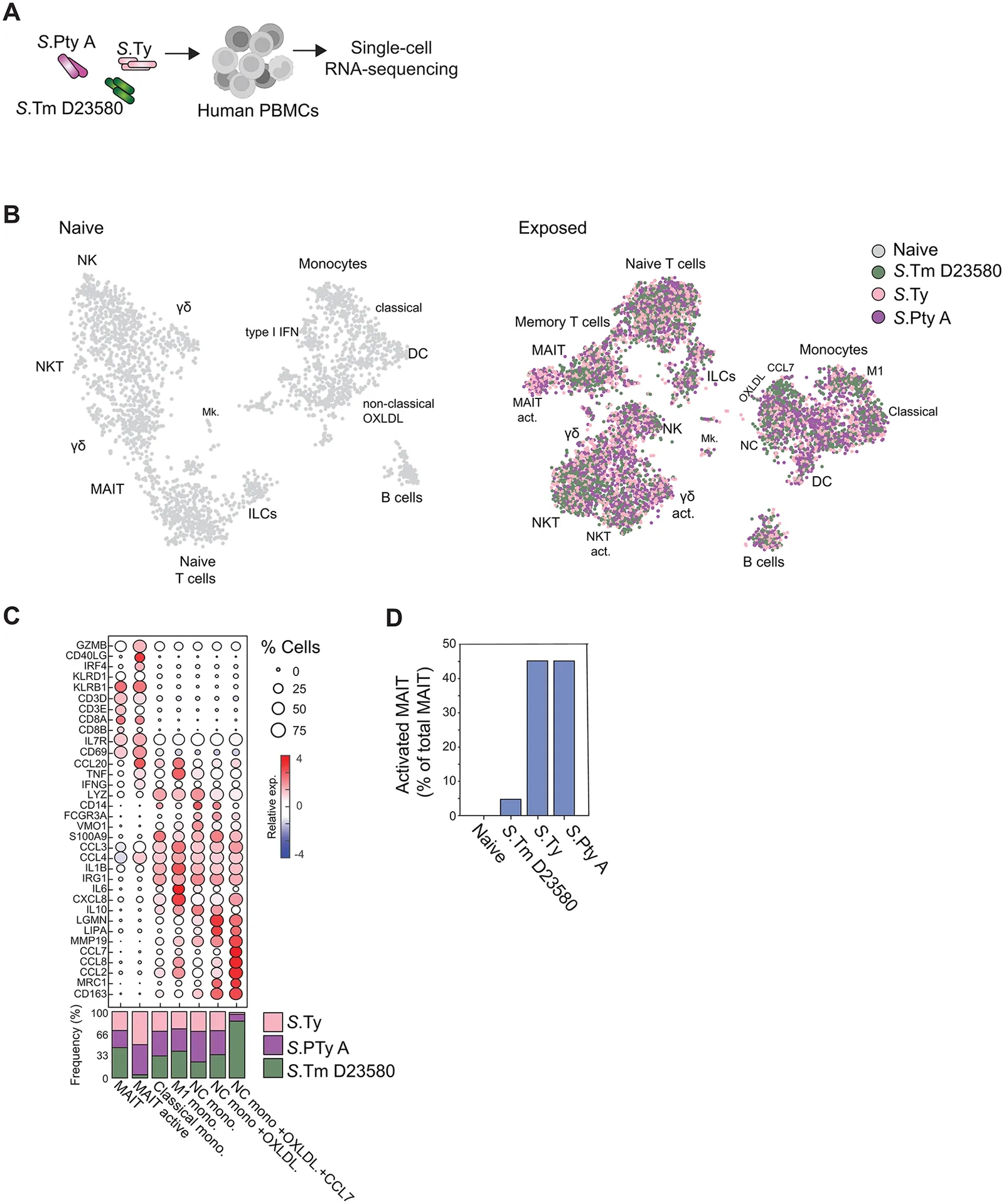
scRNA sequencing revealed serovar-specific MAIT cell activation. **(A)** Experimental outline: PBMCs from a healthy donor were either left uninfected (naïve), or infected with *S*.Ty, S.Pty A or D23580 and analyzed by scRNA-seq. **(B)** t-SNE representation of naive or infected PBMCs, with the indicated immune cell populations. Colors represent infecting serovar. **(C)** Dot-plot of marker genes used to identify total and activated MAIT cell populations and monocyte subsets. Dot size represents the percentage of cells in a subset expressing a given gene, and dot color indicates relative expression levels (legend at the right). **(D)** Frequency of activated MAIT cells (expressing TNF, IFNγ and CCL20) of total MAIT.

It was previously shown that gut-restricted *S*.Tm strains can induce robust activation of MAIT cells [13,28]. However, infection with *Salmonella* isolates within ST313 lineage 2 (*e.g.,* D23580) exhibits markedly reduced MAIT cell activation [13], though underlying mechanisms are not understood. We next focused on MAIT cell activation by different *Salmonella* serovars and isolates.

### Flow cytometry analysis confirmed lineage- specific MAIT cell activation

To further elucidate serovar-specific activation of MAIT cells, we performed flow cytometry analyses of MAIT cells in infected PBMCs. Following infection at MOI 1 by *S.*Ty, *S.*Pty A, or *S.*Tm D23580, samples were processed and stained for canonical MAIT cell surface markers CD3 ⁺ CD161 ⁺ Vα7.2⁺ [16,28]. MAIT cell activation was assessed by intracellular staining for IFNγ^+^ and TNFα^+^ (S2A Fig, with activation defined as expression of IFNγ, TNFα, or both). In agreement with the scRNA-seq data, total MAIT cell frequencies did not significantly differ across conditions (S2B Fig). Activation of MAIT cells was markedly enhanced following *S.*Ty or *S.*Pty A infection compared to D23580 infection (Fig 2A), confirming serovar-specific activation patterns.

**Fig 2:**
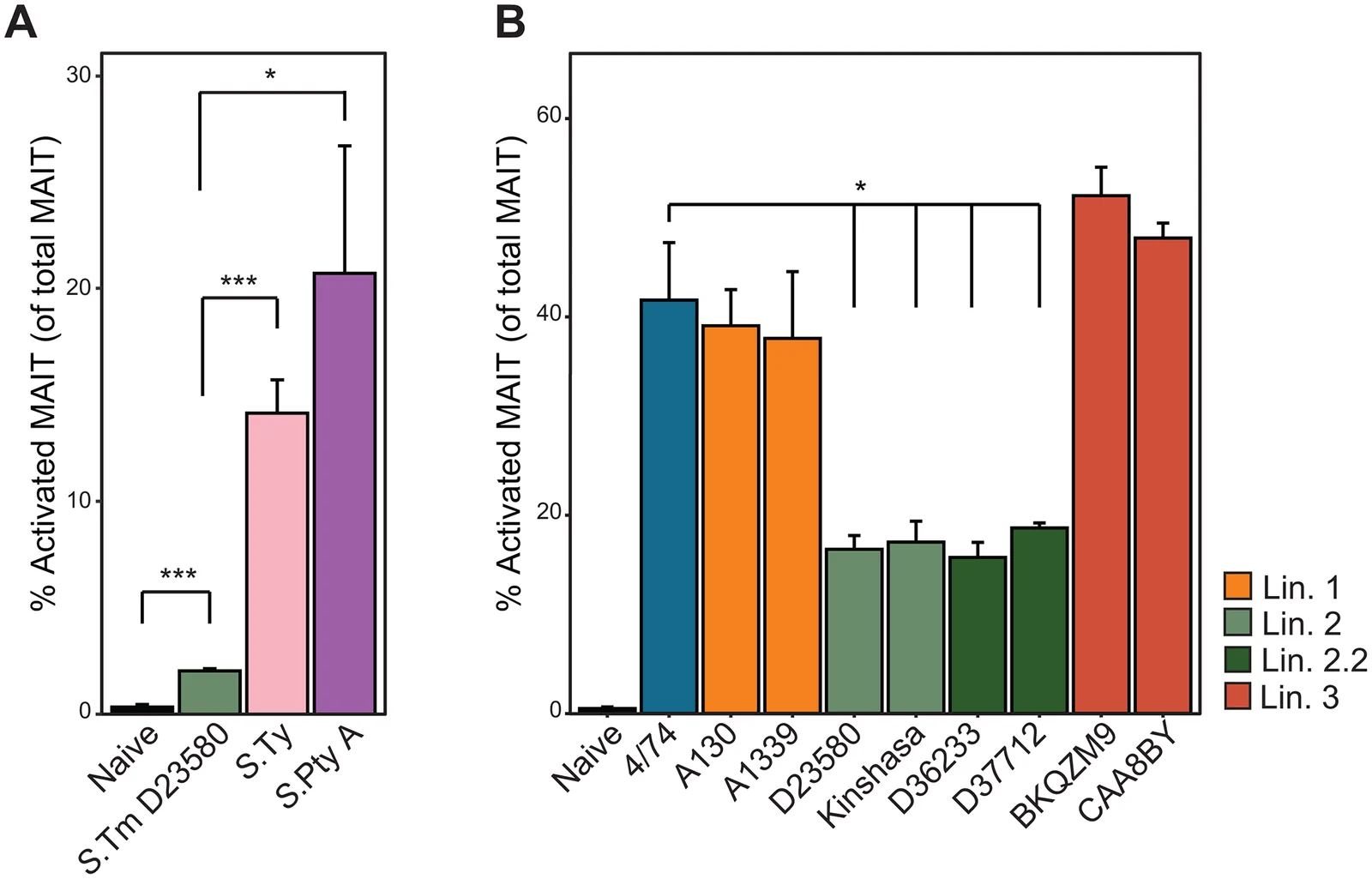
Flow cytometry analysis confirmed serovar- and lineage- specific MAIT cell activation. **(A)** Bar plot representing flow cytometry analysis of activated MAIT cells (from total MAIT cells) from naïve PBMCs or PBMCs infected with S.Ty, S.Pty A or D23580. **(B)** Bar plot representing flow cytometry analysis of activated MAIT cells (from total MAIT cells) from naïve PBMCs or PBMCs infected with serovar Typhimurium ST19 (4/74), ST313 lineage 1 (L1; A130, A1339), lineage 2 (L2; D23580, Kinshasa), lineage 2.2 (L2.2; D36233, D37712), and lineage 3 (L3; BKQZM9, CAA8By). MAIT cell activation is defined as positive for IFNγ, TNFα or both. Statistics: Student t-test, 3 replicates per condition (*) *p* < 0.05; (**) *p* < 0.001; (***) *p* < 0.0005.

To further elucidate MAIT cell activation across genetically related *S.*Tm isolates, PBMCs were infected with ST313 isolates derived from lineage 1 (A130, A1339), lineage 2 (Kinshasa), lineage 2.2 (D23580, D36233, D37712), and lineage 3 (BKQZM9, CAA8By), in addition to the genetically similar non-invasive 4/74 strain [10,36]. Total MAIT cell frequencies were similar and ranged from 3–5% of total T cells across all conditions (S2C Fig). MAIT cell activation was significantly reduced in response to infection by ST313 lineage 2 and 2.2 strains compared to all other strains examined (Fig 2B). These findings underscore a reduced MAIT cell response elicited by specific phylogenetic subgroups within ST313, as was previously demonstrated [13].

### Bacterial factors mediated strain-specific MAIT cell activation

To explore bacterial factors that underlie differences in MAIT cell activation, we focused our studies on the genetically related *S.*Tm D23580 and 4/74 strains. As *S*.Tm virulence genes are expressed differentially between growth phases [37], we compared MAIT cell activation upon infection of PBMCs with *S.*Tm strains grown to logarithmic and stationary phase. Flow cytometry analysis of infected cells revealed that the proportion of MAIT cells within the T cell population was similar between log and stationary phases in naïve, 4/74, and D23580 conditions, with a significant difference between 4/74 and D23580 in the stationary phase (S3A Fig). Interestingly, in both log and stationary growth phases 4/74 elicited significantly higher MAIT cell activation compared to D23580 (Fig 3A). To test whether the observed differences in MAIT cell activation were mediated by antigen presentation on MR1 or antigen-independent [21], we treated infected PBMCs with ɑ-MR1 antibodies or with IgG2ɑ isotype as a control and infected with D23580 and 4/74 strains. MR1 blockade abrogated MAIT cell activation in response to 4/74 (Fig 3B) with no change in the proportion of MAIT cells within the T cell population (S3B Fig), confirming that MAIT cell activation in the context of *Salmonella* infection of PBMCs is MR1-dependent.

**Fig 3:**
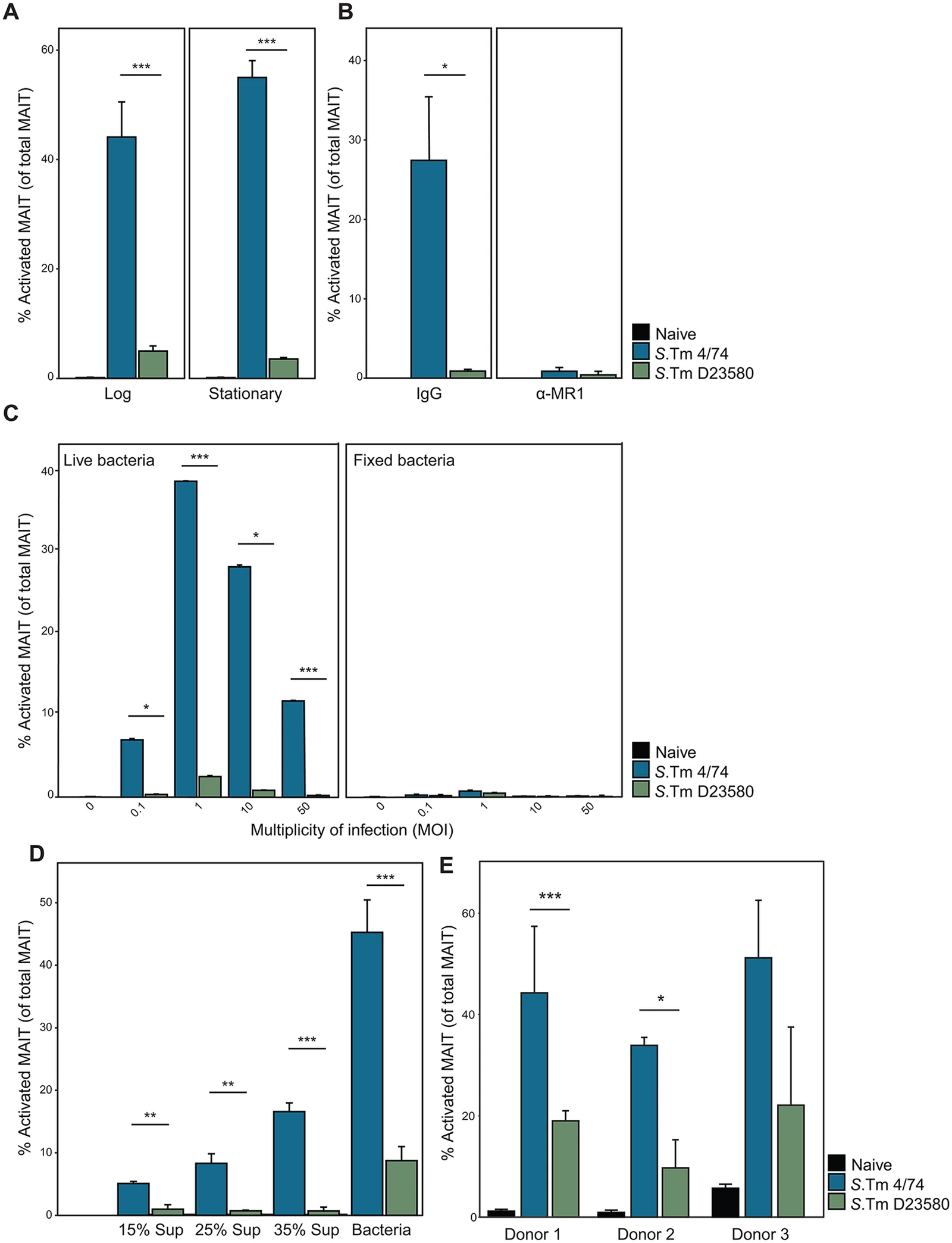
Bacterial factors mediated strain-specific MAIT cell activation. **(A)** Flow cytometry analysis of activated MAIT cells from naive PBMCs or PBMCs infected with log or stationary 4/74 (blue) or D23580 (green). **(B)** Flow cytometry analysis of activated MAIT cells from PBMCs treated with an aMR1 blocking antibody or IgG isotype control, and either left uninfected (naïve) or infected with 4/74 and D23580. **(C)** Flow cytometry analysis of activated MAIT cells from naive PBMCs or PBMCs infected with live or PFA-fixed 4/74 and D23580, at MOI of 0.1, 1, 10, or 50. **(D)** Flow cytometry analysis of activated MAIT cells from PBMCs treated with bacterial supernatants from 4/74 (blue) and D23580 (green) in concentrations of 15%, 25%, or 35% of total culture volume. Infection with live bacterial strains was used as positive control. MAIT cell activation is defined as positive for IFNγ, TNFα or both. **(E)** Flow cytometry analysis of activated MAIT cells of PBMCs from 3 different individuals (n = 3 per donor), either left uninfected (naïve) or infected with 4/74 and D23580. Statistics: Student T-test. (*) *p* < 0.05; (**) *p* < 0.001; (***) *p* < 0.0005.

Next, we tested whether MAIT cell activation requires live bacterial infection. We exposed PBMCs to 4/74 and D23580, either live or paraformaldehyde (PFA)–fixed, at increasing MOIs (0.1, 1, 10, 50). Robust activation of MAIT cells was observed exclusively in PBMCs infected with live 4/74 strain (Fig 3C). PFA-fixed bacteria failed to elicit MAIT cell responses, irrespective of MOI. At MOI 50, there was a significant reduction in total MAIT cells in PBMCs infected with live 4/74, as well as fixed 4/74 and D23580 (S3C Fig), probably due to the high bacterial burden and overwhelming activation [19,38]. Next, we exposed PBMCs to sterile-filtered culture supernatants derived from 4/74 or D23580. While the proportion of MAIT cells within the T cell population was similar across all conditions (S3D Fig), filtered 4/74 bacterial supernatants induced a dose-dependent MAIT cell activation, whereas bacterial supernatants from D23580 cultures did not activate MAIT cells (Fig 3D). Finally, we infected PBMCs isolated from three individuals with *S*.Tm 4/74 or *S*.Tm D23580 (Fig 3E). While the magnitude of MAIT activation varied between donors, *S*.Tm 4/74 elicited a stronger MAIT response than *S*.Tm D23580 in every donor demonstrating a consistent strain-dependent effect.

Collectively, these findings suggest that MAIT cell activation by 4/74 *S*.Tm is dependent upon the active biosynthesis of MR1-binding MAIT cell antigens. These can either be produced during live bacterial infection or secreted into the supernatant. We next sought to analyze the MAIT cell-stimulatory ligands that underlie differential activation between D23580 and 4/74.

### Genetic perturbations of *Salmonella rib* enzymes revealed strain-specific growth inhibition

MAIT cell activation is mediated by presentation of specific ligands derived from the RF biosynthesis pathway (Fig 4A), mainly 5-A-RU and DMRL [39,40]. *Salmonella* species actively express *rib* genes encoding enzymes involved in RF biosynthesis and produce intermediates that activate MAIT cells. The coding and regulatory sequences of *rib* enzymes are mostly conserved between D23580 and 4/74 [10,41]. Expression levels of enzymes were found to be comparable between 4/74 and D23580, excluding RibB, which is overexpressed in D23580, although the significance of the upregulation to infection is unclear [13]. Since *rib* enzymes are essential [42], we turned to construct an inducible knockdown system using CRISPRi-dCas9 hypomorphs [43] to test their involvement in the differential MAIT activation phenotype. We designed single guide RNA (sgRNA) for each *rib* gene involved in the production of MAIT cell-activating ligands: *RibA*, *RibB*, *RibD*, *RibH* and *RibE*, in addition to a control strain with a scramble sequence (control) and inserted them into 4/74 and D23580 genomes. We verified inducible gene downregulation using quantitative real-time PCR (qRT-PCR) of 4/74 and D23580 grown in the absence or presence of IPTG (Fig 4B). Notably, when *RibA*, *RibH* and *RibE* were downregulated, *RibB* expression was elevated. This is likely due to the effect of knockdown in the pathway on the production of downstream compound FMN and the subsequent release of *RibB* repression by the FMN Riboswitch [25,44,45]. A similar effect was reported following the deletion of the bacterial FMN-Riboswitch in *E.coli* [46].

**Fig 4:**
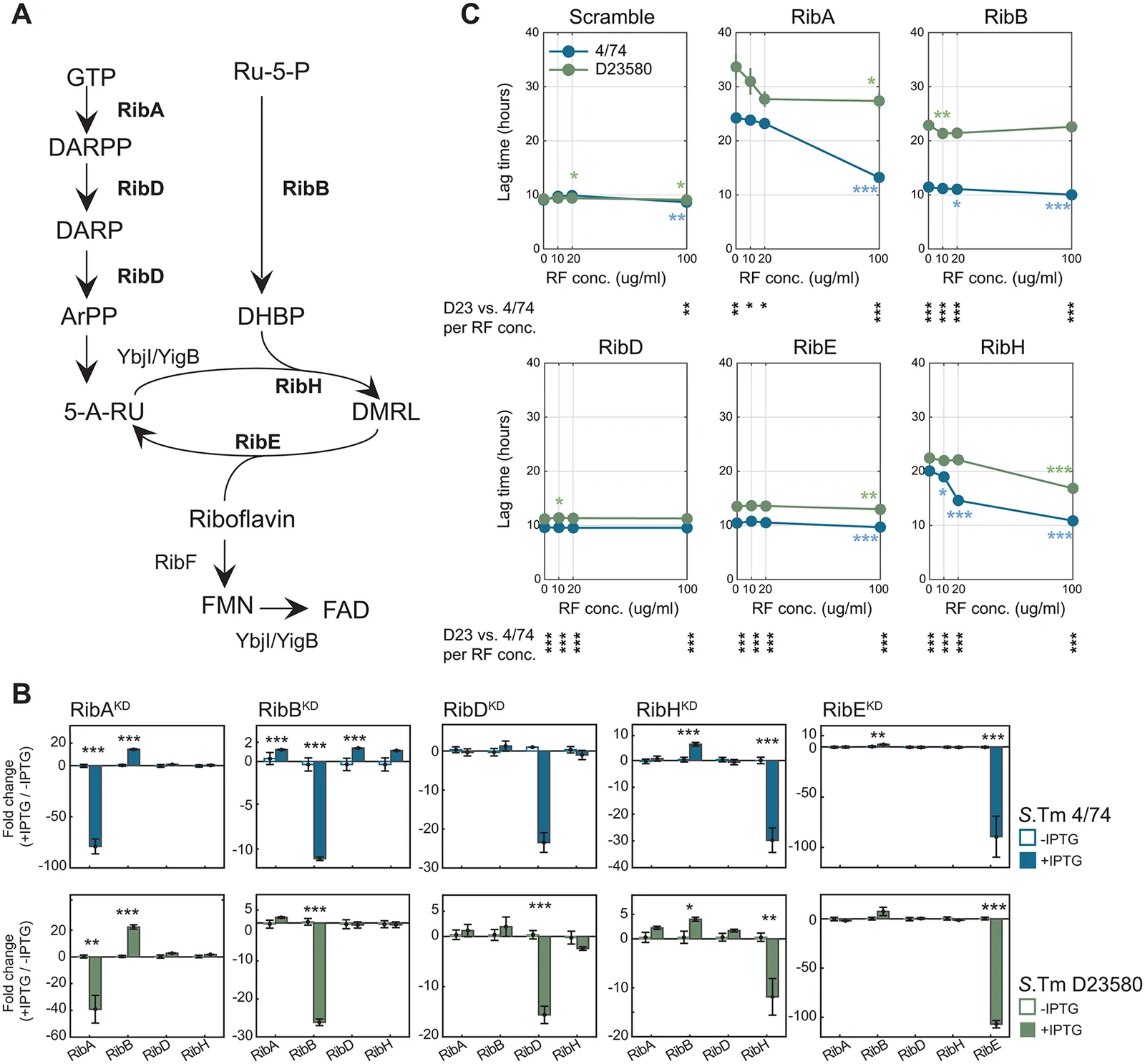
Genetic perturbations of *Salmonella rib* enzymes revealed strain-specific growth inhibition. **(A)** Schematic representation of the RF biosynthesis pathway in *Salmonella*. Indicated in bold are enzymes involved in the pathway that were targeted by CRISPRi. **(B)** RNA was isolated from cultures of the indicated *rib* hypomorphs of 4/74 (blue) and D23580 (green). Presented is qRT-PCR with primers targeting *rib* genes (X-axis) as fold change of expression relative to the mean without IPTG (Y-axis). **(C)** Lag times of the indicated *rib* hypomorphs in minimal media supplemented with the indicated RF concentrations. Colored stars indicate the significance relative to no RF within each strain. Statistics: two-sample t-test (*) *p* < 0.05; (**) *p* < 0.001; (***) *p* < 0.0005.

We next assessed the functional consequences of perturbation to the RF pathway on bacterial growth. Rib^KD^ hypomorphs were cultured in M9 minimal media (MM) and supplemented with 0.4% glucose as the sole carbon source. Cultures were grown in the presence of IPTG and exogenous RF at concentrations of 0, 10, 20 or 100 µg/ml for 36 hours, and growth was measured by OD600 (S4A Fig), or on agar for colony forming units (CFU; S4B Fig). To quantify growth, we calculated the lag time of each strain (Fig 4C) and verified similar growth of scramble controls in 4/74 and D23580 strains, independent of exogenous RF. RibA^KD^ exhibited delayed lag time, which was more pronounced in D23580 compared to 4/74. In both strains, a dose-dependent decrease in lag time was observed upon RF addition to the media. In RibB^KD^, we measured increased time to lag in D23580 that was not recovered by RF at any concentration. RibD^KD^ and RibE^KD^ had minor but significant effects on lag time in 4/74 and D23580 strains. RibH^KD^ exhibited delayed time to lag in D23580 and 4/74, with a dose-dependent growth restoration more pronounced in 4/74 compared to D23580. These results suggested that D23580 hypomorphs were significantly more delayed in lag time compared to 4/74 hypomorphs.

As we found reduced growth and no recovery by exogenous RF in RibB^KD^ in D23580, we introduced a RF transporter, RibN, from *Rhizobium leguminosarum* [47] into the RibB^KD^ hypomorph, to test if effective uptake of exogenous RF restores growth. D23580 RibB^KD^, with marked growth deficiency, fully recovered with exogenous RF (10 µM to 250 µM) when expressing RibN (S4C Fig). This result indicates that D23580 can recover growth by efficient exogenous RF uptake.

### Strain-specific RibH^KD^ and RibE^KD^ effects on MAIT cell activation

We turned to examine our hypothesis that manipulation of the RF biosynthesis enzymes in 4/74 and D23580 could underlie differential MAIT cell activation. We grew *rib* hypomorphs in LB in the absence or presence of IPTG to induce gene knockdown. We infected PBMCs with the different strains and measured MAIT cell activation by flow cytometry (Fig 5A). We verified that scramble controls had similar effect on MAIT cell activation in the presence or absence of IPTG. Knockdown of RibA, RibB, and RibD did not significantly impact MAIT cell activation, comparing between without or with IPTG induction. Interestingly, induction of RibH^KD^ in D23580 led to significantly increased MAIT cell activation, compared to no IPTG, while in 4/74 there was not significant impact of RibH^KD^. In contrast, infection with 4/74 RibE^KD^ hypomorph induced by IPTG resulted in reduced MAIT cell activation, compared to no induction, while in D23580 there was no significant impact on RibE^KD^ without or with IPTG. As we found that bacterial supernatants stimulate MAIT cell activation differentially between 4/74 and D23580, we tested MAIT cell stimulation by supernatants of RibH^KD^ bacterial hypomorphs grown without or with IPTG. We measured significantly elevated MAIT cell activation in response to supernatants of both RibH^KD^ bacterial hypomorphs in 4/74 and D23580 (Fig 5B). We next tested if MAIT cell ligands might be exported by a known RF transporter, RibN. While primarily functioning as importers of RF to scavenge the nutrient from the environment, RF transporters can also catalyze bidirectional translocation, potentially facilitating RF export [48]. We reasoned that if RibN expression in D23580 will induce MAIT cell activation similar to 4/74, it will suggest that MAIT cell ligands may be differentially exported by these strains. We infected PBMCs with the strains carrying RibN or an empty vector, and measured MAIT cell response to infection. MAIT cell activation remained unchanged irrespective of expression of the transporter (S5A Fig), suggesting that either RibN does not export MAIT cell ligands or that active secretion is not the mechanism of differential MAIT cell activation by culture supernatants.

**Fig 5:**
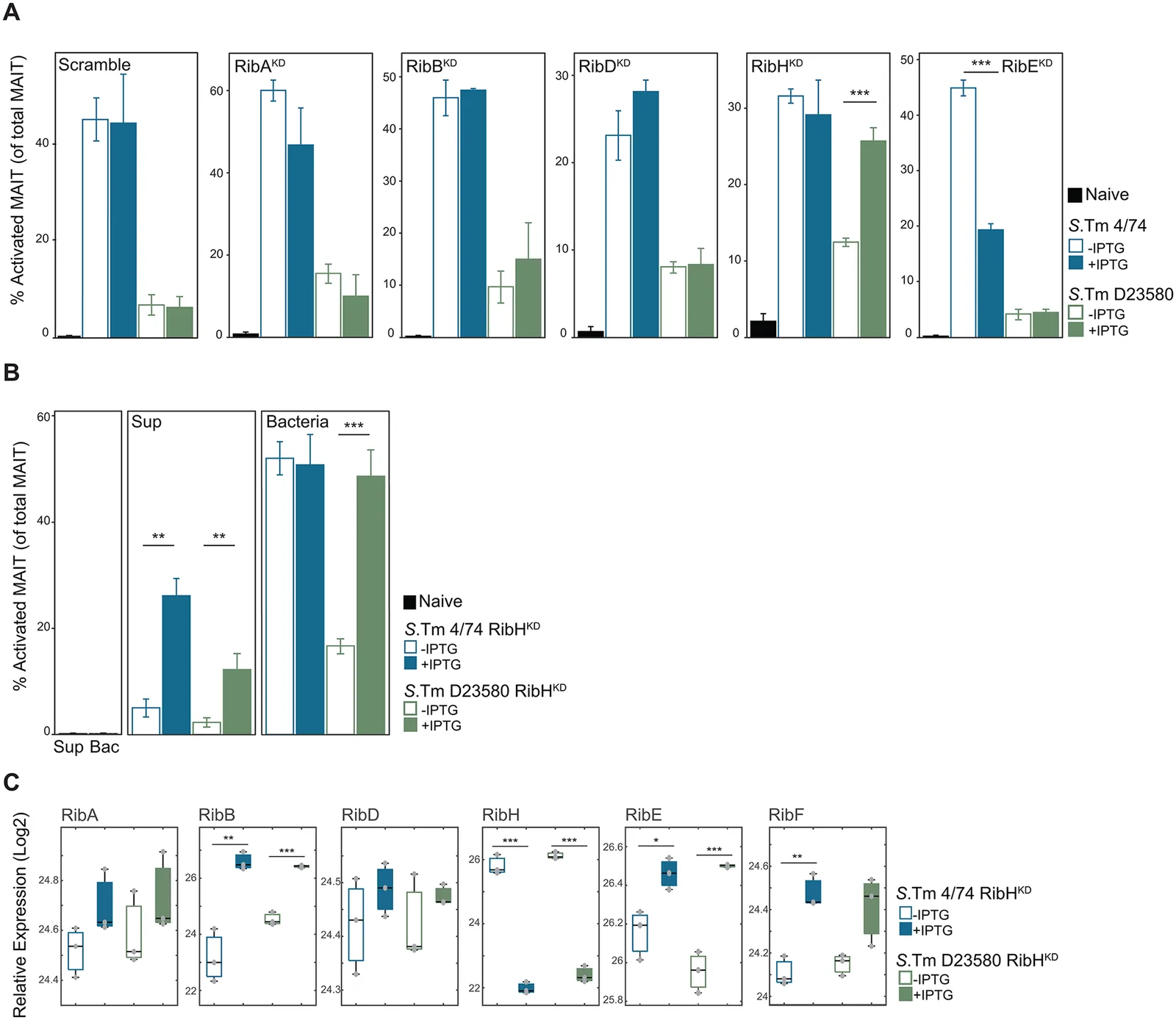
Strain-specific RibH^KD^ and RibE^KD^ effects on MAIT cells activation. **(A)** Flow cytometry analysis of activated MAIT cells from naïve PBMCs or PBMCs infected with the indicated *rib* hypomorphs of 4/74 (blue) and D23580 (green) in the presence or absence of IPTG. **(B)** Flow cytometry measurements of MAIT cell activation from PBMCs stimulated with bacterial supernatants or live infection with RibH^KD^ in 4/74 (blue) and D23580 (green). MAIT cell activation is defined as positive for IFNγ, TNFα or both. **(C)** Boxplots presenting the expression of *rib* enzymes from proteomics analysis of RibH^KD^ in 4/74 (blue) and D23580 (green). Statistics: two-sample t-test (*) *p* < 0.05; (**) *p* < 0.001; (***) *p* < 0.0005; all significant results are with q-value<0.1 (10%FDR).

To study bacterial pathways that are modified in RibH^KD^ that may mediate the effects we observed on bacterial growth and MAIT cell activation, we analyzed 4/74 and D23580 RibH^KD^ hypomorphs by proteomics (S5B-C Fig, S1 Table). We measured a significant 13-to-14-fold decrease in RibH protein abundance in RibH^KD^ hypomorphs of both strains comparing bacteria grown without or with IPTG (Fig 5C). Consistent with our qRT-PCR measurements, RibB levels were significantly increased following RibH knockdown. Further analysis of proteins affected by RibH knockdown in both strains revealed an elevated expression of *thi* proteins (thiC/D/I/G/E) involved in Thiamine (vitamin B1) metabolism [49] (S5D Fig). Thiamine upregulation, necessary for the generation of NAD and NADP cofactors, suggests a shift in cellular energy production pathways in the absence of FMN and FAD. D23580 RibH^KD^ also expressed elevated levels of *ent* proteins (entA/B/C/E/F/H) required for iron scavenging in low-iron environments [50] (S1 Table). This could potentially suggest that D23580 better relays iron redox of low-energy substrates like H_2_ or nitrate when nutrients needed for aerobic respiration are limited [51]. A significant decrease was detected in proteins associated with the TCA cycle (sucC/D, sdhA/B/C/D, frdA/B/C/D) [52], glyoxylate metabolism (acnA/B, ttbB) and sulfur metabolism (cysA/C/D/H/J/P, sbp) [53,54] as well as virulence proteins such as the SPI-1 regulon (inve/G/J, sopB, prgH/K, hilD) [55,56]. In 4/74 RibH^KD^ an additional reduction was detected in proteins involved in alanine, aspartate and glutamate metabolism (asnA/B, carA/B, pyrB/I) [57,58] as well as lysine, valine, leucine and isoleucine degradation (fadA/B/J, DLD) [59]. Histidine metabolism was strongly downregulated in RibH^KD^ in both strains. Given that purine synthesis, required for riboflavin biosynthesis (Fig 4A), is regulated by histidine availability, this reduction may reflect altered precursor availability or a broader adaptation to reduced flavin cofactor levels. We did not detect pathways or other proteins implicated in MAIT cell activation. However, our proteomics data suggested that the strains utilize different mechanisms to resolve supposed FMN/FAD deficiency resulting from RibH silencing, possibly resulting in altered metabolism between the strains, in addition to differential MAIT cell activation.

### RibH was a key determinant of differential strain-specific MAIT activation

Our observations of altered MAIT cell activation in PBMCs in response to RibH^KD^ and RibE^KD^ hypomorphs suggested a shift in the respective repertoires of MAIT cell -activating ligands. To further explore this, we turned to a co-culture system using the human MR1-restricted MAIT cell clone D426-G11 and the bronchial epithelial BEAS-2B cell line. D426-G11 is activated with high sensitivity to riboflavin pathway metabolites, presented by the MHC class I-related protein MR1 on the BEAS-2B cell line [60]. We infected these cells with WT, Scramble, RibH^KD^ or RibE^KD^ hypomorphs of both D23580 and 4/74, and measured MAIT cell activation by IFNγ enzyme-linked immunospot (ELISPOT) assay (Fig 6A). Phytohemagglutinin (PHA) was used as a positive control as it activates MAIT cells irrespective of MR1, and MAIT cells co-cultured with BEAS-2B MR1^KO^ or without APCs were used as negative controls (S6A Fig). This more reductionistic model system confirmed our observations made in PBMCs. As in the flow cytometry assay, we observed a significant increase in IFNγ production in response to RibH^KD^ in D23580, while supernatants of RibH^KD^ in both 4/74 and D23580 increased IFNγ levels. Additionally, IFNγ production was reduced upon induction of RibE^KD^ in the 4/74 strain, similar to our results in PBMCs. These functional differences could not be explained by differential bacterial uptake (S6B Fig).

**Fig 6:**
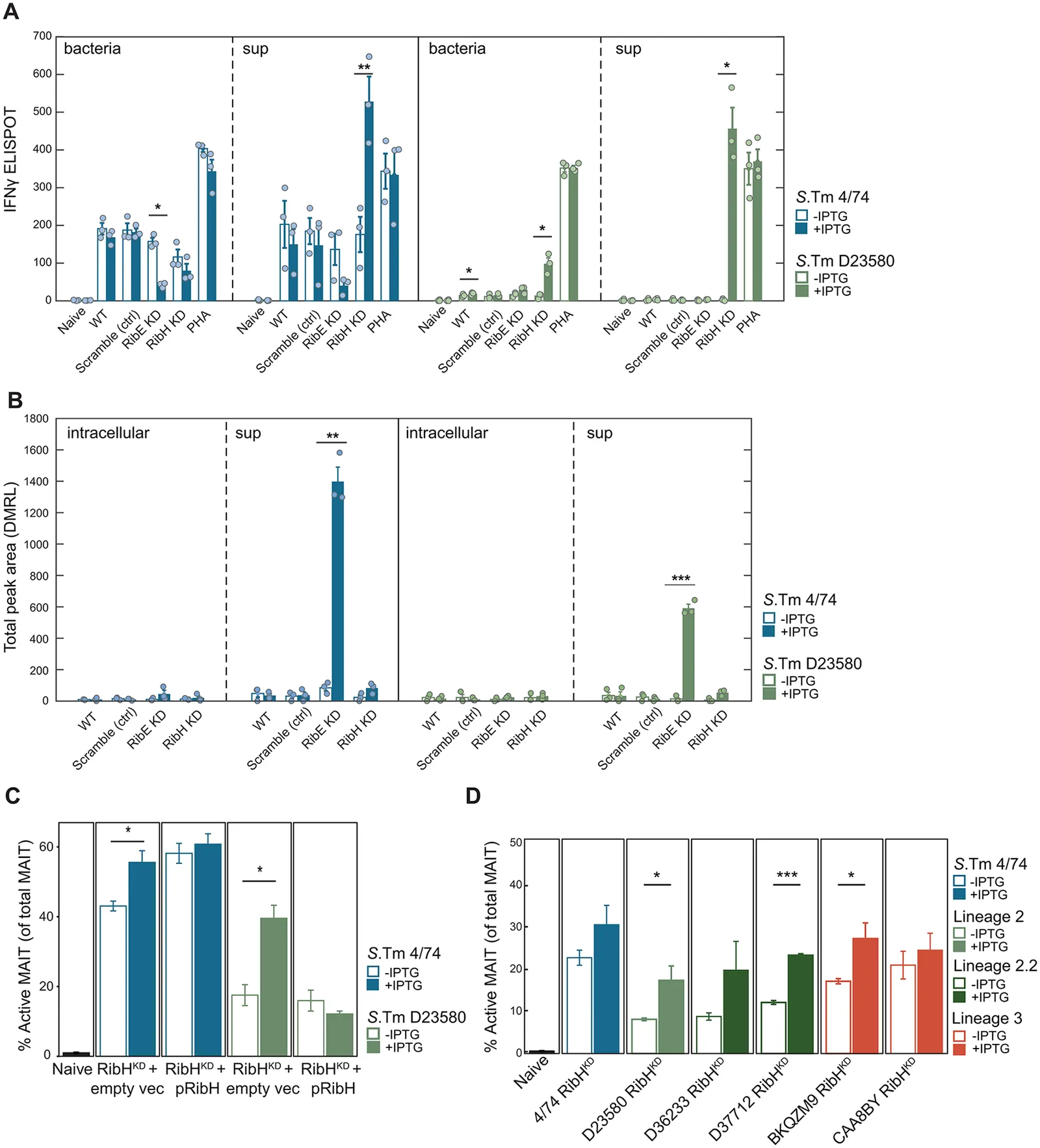
RibH was the main source for differential strain-specific MAIT cell activation. **(A)** BEAS-2B cells were infected with WT, scramble, RibH^KD^, or RibE^KD^ hypomorphs of strains D23580 or 4/74 before co-culture with the human MAIT cell clone D426-G11. MAIT cell activation was measured by IFN-γ enzyme-linked immunospot (ELISPOT) assay. Phytohemagglutinin (PHA) was added as positive control. Data are pooled from three independent experiments, and each dot represents the mean of technical duplicates. **(B)** DMRL in culture supernatants of RibE^KD^ and RibH^KD^ in 4/74 and D23580 was quantified using DMRM. Quantification was performed using chromatographic peak area of the most stable transition ion (m/z 327.1 → 193) of DMRL. **(C)** Flow cytometry analysis of activated MAIT cells from naïve PBMCs or PBMCs infected with RibH^KD^ 4/74 (blue) or D23580 (green) carrying a plasmid expressing RibH (pRibH) or an empty vector (empty vec). **(D)** Flow cytometry analysis of activated MAIT cells from naïve PBMCs or PBMCs infected with RibH^KD^ in 4/74, D23580, D36233, D37712, BKQZM9 and CAA8BY. MAIT cell activation is defined as positive for IFNγ, TNFα or both. Statistics: paired t-test between with and without IPTG for **(A-B)**, Student t-test for **(C-D)** (*) *p* < 0.05; (**) *p* < 0.001; (***) *p* < 0.0005.

We next examined how these genetic disruptions affected metabolic intermediates within the RF biosynthetic pathway. To assess this, we measured DMRL, FMN, FAD and riboflavin in both supernatants and cellular material using LC-MS/MS Multiple Reaction Monitoring (MRM) metabolomics (Fig 6B and S6C Fig). 5-A-RU was not assessed due to its instability, which limits its detection by mass spectrometry [61]. We found that DMRL levels in the supernatants of RibE^KD^ mutants were highly elevated compared to scramble controls. This is consistent with the role of RibE in RF biosynthesis which converts two molecules of DMRL into one molecule of RF and one molecule of 5-A-RU. As DMRL was not detected in bacterial lysate but only in supernatants of RibE^KD^, it is tempting to speculate that excess DMRL is secreted. Furthermore, we measured significantly reduced levels of RF, FAD and FMN in RibH^KD^ in the 4/74 but not the D23580 bacteria pellets (S6C Fig), indicating reduced flux in the RF biosynthesis pathway, which may influence the availability of upstream intermediates such as 5-A-RU.

To test whether RibH^KD^ directly impacts MAIT cell activation, we complemented RibH^KD^ hypomorphs by introducing a plasmid constitutively expressing the native RibH enzyme (pRibH) or an empty vector (control). We validated by qRT-PCR that the expression of *RibH* was significantly upregulated in bacteria transfected with pRibH compared to bacteria with an empty vector (S6D Fig). To assess the impact on MAIT cell activation, PBMCs were infected with both RibH^KD^ hypomorphs carrying either pRibH or an empty vector. The proportion of MAIT cells within the T cell population was similar between without or with IPTG (S6E Fig). In 4/74, bacteria carrying pRibH elicited a similar MAIT cell activation comparing no induction and IPTG induction. PBMCs infected with RibH^KD^ D23580 carrying pRibH had significantly reduced MAIT cell activation, compared to infection with RibH^KD^ carrying the empty plasmid (Fig 6C). We then tested the effects of differential MAIT cell activation on infection outcome. We infected PBMCs with RibH^KD^ and RibE^KD^ hypomorphs in D23580 and 4/74 strains and found only a minor impact on intracellular bacterial survival, measured by CFU (S6F-G Fig).

Finally, we introduced RibH^KD^ CRISPRi into ST313 strains from lineage 2.2 (D36233 and D37712), and lineage 3 (BKQZM9 and CAA8BY). We infected PBMCs with these strains grown in the absence or presence of IPTG. RibH^KD^ exhibited enhanced MAIT cell activation in response to infection with most strains, most prominently lineage 2 and 2.2 (Fig 6D, S6H Fig). These findings further support the general role of RibH expression in strain and lineage-specific modulation MAIT cell-mediated innate immune responses during infection.

## Discussion

MAIT cell activation is tightly linked to microbial RF biosynthesis [21]. This is illustrated by RF pathway deletion mutants in *Lactococcus lactis* [28], *Escherichia coli* [62], and *Streptococcus pneumoniae* [59], where loss of RF biosynthesis abolishes MAIT cell activation, and the observation that RF pathway metabolites are required for MR1-dependent antigen presentation. In this study, we demonstrated that lumazine synthase (RibH) functions as the main metabolic activation node for MAIT cells during *Salmonella* infection, with activity that differs across *Salmonella* strains and ST313 lineages. We observed that *S.*Tm ST313, lineages 2 and 2.2, induce minimal MAIT cell activation, in contrast to lineages 1 and 3 and 4/74, as was previously demonstrated [13]. To gain mechanistic insight, we engineered conditional knockdowns using CRISPRi to knockdown the expression of enzymes in the RF pathway in 4/74 and D23580. We observed that disrupting the RF pathway at an early stage (*RibA*, *RibB* or *RibD*) had no significant impact on MAIT cell activation in both strains. Knocking down RibH in D23580 resulted in increased MAIT cell activation, possibly due to accumulation of 5-A-RU, which can react with methylglyoxal to form the potent MAIT cell–activating ligand 5-OP-RU [63]. We measured a similar impact of RibH^KD^ in ST313 lineage 2 and 2.2 strains. In contrast, RibH^KD^ in strains that activate MAIT cells naturally (*e.g.,* 4/74 and ST313 lineage 1 and 3) had only a minor impact. This presents an intriguing paradox, as the RF biosynthesis enzymes producing MAIT cell activating ligands are conserved between ST313 lineages with identical protein sequence. Comparative genomic and transcriptomic analyses of 4/74 and D23580 revealed near-identical sequences and expression profiles, with the only difference being a silent SNP in the coding region of *ribD* that does not affect overall RibD expression [13]. Notably, RibB expression is significantly elevated in D23580 compared with 4/74, despite identical coding and regulatory sequences [13]. However, RibB knockdown did not alter MAIT cell activation in our study, arguing against a direct role for RibB abundance alone.

RibH knockdown was shown as a central node also in *Mycobacterium tuberculosis* and avirulent *Mycobacterium smegmatis*, impacting differential MAIT cell activation [40] and growth recovery [64] between the two species. These results, together with the current study in *Salmonella,* suggest that RibH^KD^ and RibE^KD^ may represent a global bacterial node regulating MAIT cell activation. In *Mycobacterium tuberculosis,* it was demonstrated that a flux of 5-A-RU towards deazaflavins at the expense of lumazine compounds reduced the MAIT cell response to the bacteria [40]. Our results of the differential impact of RibH^KD^ and RibE^KD^ in *Salmonella* could be due to analogous altered flux through the RF pathway between ST313 isolates, although parallel enzymes for deazaflavin production are not present in *Salmonella*. Indeed, our metabolomics measurements indicated reduced levels of RF biosynthesis end products (RF, FMN and FAD) in 4/74 RibH^KD^ compared to D23580 RibH^KD^, consistent with altered metabolic pathway flux. As 5-A-RU is extremely unstable and challenging to detect experimentally by metabolic profiling [18,20,28], further analyses will be required to directly study the abundance of this ligand between the *ribH* hypomorphs. The accumulation of the DMRL intermediate in RibE^KD^ supported altered metabolic flux downstream of RibE. Our proteomics analyses further suggested that the 4/74 and D23580 strains utilize different mechanisms to resolve the presumed RF deficiency caused by RibH^KD^. Finally, RibB overexpression in D23580 could indicate reduced intracellular FMN levels, which relieve repression of the FMN-responsive RibB riboswitch [45], consistent with altered RF pathway regulation. Taken together, this evidence suggests differences in metabolic processing that could underlie strain-specific MAIT cell activation despite genetic similarity. Our results indicate that knockdown of RibE, which converts two DMRL molecules to 5-A-RU and the non-immunogenic RF, resulted in DMRL accumulation in the supernatant of both strains. Although DMRL is a weakly stimulatory MAIT antigen itself [26], its accumulation in RibE^KD^ reduced the MAIT cell response in 4/74 and did not alter MAIT cell responses in D23580, which complicates our understanding of its role in MAIT cell activation during *Salmonella* infection.

We propose that human PBMC-based infection models offer a physiologically relevant system for investigating immune responses against human-associated pathogens. MAIT cells in particular, constitute approximately 3–10% of circulating T cells in human blood and gut, but are markedly less abundant in mice (~0.1%) [15], raising the possibility that gut infection models of mice may not recapitulate the physiologically role of MAIT cells during *Salmonella* infection. As MAIT cells are enriched in the murine lung [65], models of intranasal *Salmonella* infection have suggested a role for MAIT cells in controlling pulmonary infection [23]*.* However, because the natural route of *Salmonella* infection is oral, via the gastrointestinal tract, the relevance of these findings remains unclear. In contrast, *in vivo* studies of human typhoid challenge model suggested a role for MAIT cells in mounting an efficient immune response [19], and a significant increase of activated MAIT cells in individuals that developed enteric fever [29].

PBMCs are a primary and heterogeneous immune cell population. While *ex vivo* infection of human PBMCs provide access to primary human immune cells and preserve interactions among multiple immune populations, they do not fully recapitulate the complexity of infection *in vivo*. PBMCs lack tissue resident immune cells and recruited immune cells that shape the local response to infection. Furthermore, inter-individual variation among human donors introduces additional heterogeneity, although it also provides an opportunity to identify immune responses that are conserved across individuals, as we have shown here. Thus, future studies in complementary tissue-based or in vivo models will be important to establish the physiological relevance of these findings.

We hypothesize that during infection of PBMCs, live bacteria are taken up by phagocytic monocytes and presented by MR1 to MAIT cells. Indeed, blocking MR1 in PBMCs completely abolished MAIT cell activation during *Salmonella* infection. We detected similar results with a more controlled *in vitro* co-culture MAIT cells system. However, while infection with live bacteria elicited heightened MAIT cell activation compared to bacterial supernatant in PBMCs, in the engineered system the bacterial supernatants were a more potent source of MAIT cell antigens. One plausible explanation for this difference is that the bronchial epithelial cells are non-phagocytic and thus do not efficiently take up live bacteria but may take up MAIT cell ligands from culture supernatants more readily. Together with our MRM data, this raises the possibility that RF intermediates or MAIT cells ligands can be efficiently released into the extracellular environment, but the mechanism remains unknown.

In conclusion, our findings indicate differential activity of RF biosynthesis enzymes, RibH and RibE, between genetically similar ST313 iNTS strains. Although the impact of differential MAIT cell activation on *Salmonella* infection outcome remains to be determined, our data support RibH and RibE as central nodes regulating MAIT cell responses during *Salmonella* infection.

## Materials and methods

### Ethics declaration

The study was conducted in accordance with the Declaration of Helsinki and national and institutional standards and approved by the Weizmann Institute of Science Ethics Committee (number 2341–1) and the Institutional Review Board at Oregon Health & Science University (OHSU) (IRB00000186).

### Bacterial strains

All bacterial strains used in this project were originally isolated from infected individuals. *Salmonella* Typhi strain Quailes and Paratyphi A strain NVGH308 were kindly provided by Prof. Andrew Pollard’s lab, Oxford. *S.*Tm strains A130, A1339, D23580, D36233, D37712, Kinshasa, BKQZM9, CAA8BY and 4/74 were kindly provided by Prof. Jay Hinton’s lab, Liverpool University.

### PBMC extraction and treatment

Leukocyte-enriched fractions from healthy donors were obtained from the national blood bank (MDA, Israel). Blood bank samples were fully anonymized, with no donor-identifiable information, ensuring confidentiality and compliance with ethical regulations. PBMCs were isolated, aliquoted at 20 x10^6^ cells per tube, and cryopreserved as previously described [66]. For each experiment, PBMCs from a single donor were thawed, counted, and assessed for viability using ViaStain AO/PI Staining Solution (Revvity, CS2–0106). Cells were resuspended in medium consisting of RPMI 1640 with L-glutamine, supplemented with 10% heat-inactivated fetal bovine serum and 1 mM sodium pyruvate (hereafter RPMI+). PBMCs were plated at 5 x 10^5^ cells per well in U-bottom, untreated 96-well plates (Greiner Bio-One, 650185) with 3–4 wells per condition as replicates and incubated at 37°c with 5% CO2 for overnight resting prior to further treatment.

For experiments using T cell clone D426-G11, peripheral blood mononuclear cells (PBMCs) and human serum were obtained from healthy donors in Portland, Oregon, and used to expand the T cell clone (see ‘MR1T and BEAS-2B cells’ and ‘ELISPOT assays’ in the methods section). The findings in this manuscript are based on T cell clone function or on other cell line function, e.g., BEAS-2B cell line and, thus, sex and gender of PBMC donors were not taken into consideration for these experiments.

### PBMC infection

OD600 of bacterial cultures (see ‘Bacterial growth’ section in Materials and Methods) was measured and the estimated number of bacteria in each culture was calculated based on that OD_600_ of 1 corresponds to 1 x 10^9^ bacteria/mL in the early stationary phase. PBMCs were infected (3–4 wells per condition as replicates) at a multiplicity of infection (MOI) of 1, unless otherwise stated. For naïve (uninfected) samples, PBS with calcium and magnesium (hereafter, PBS+) was added instead. Samples were gently mixed by pipetting and then centrifuged for 5 minutes at 500 x g at room temperature to enhance bacterial internalization, followed by incubation for 30 minutes at 37 °C with 5% CO2. Cells were then washed twice by centrifugation for 5 minutes at 500 x g, with the supernatant discarded and the cells resuspended in RPMI+ containing 50 μg/mL gentamicin to eliminate extracellular bacteria. Infected cells were subsequently incubated at 37 °C with 5% CO2 until the designated experimental time point (CFU: 4h or 24h, FACS measurements- 20-23h post-infection).

For the MR1 blocking assay, anti-MR1 antibody (clone 26.5, BioLegend, 361102) or an IgG2a isotype control (BioLegend, 400201) was added at a final concentration of 20 μg/mL immediately before infection. In experiments using CRISPRi-dCas9 hypomorphs, samples grown overnight in the presence of IPTG were also cultured in a gentamicin-containing medium supplemented with 1 mM IPTG (Merck I6758-1G) to maintain continuous expression of the CRISPRi-dCas9 system and consistent gene knockdown.

### scRNA-seq sample preparation

PBMCs from one healthy donor were infected with *S*.Ty, *S*.Pty A or *S*.Tm strain D23580 at MOI 1. For naïve samples, PBS + was added to the cells. Four hours post-infection, cells were centrifuged at 500 x g for 5 minutes, washed with phosphate-buffered saline without calcium and magnesium (hereafter, PBS-/-), resuspended in 5 mM EDTA (Sartorius 01–862-1B) in PBS-/-, and incubated for 10 minutes at 4 °C. The cells were then centrifuged (5 min, 500 x g), supernatant was discarded, and the cells were resuspended in PBS-/- with 2% ultra-pure BSA (AM261, Thermo Fisher Scientific) and 0.01% Tween 80 (P1754-500 Sigma). For cell Hash-tagging [31], each sample was diluted to 2.5 x 10^6^ cells in 1 mL and barcoded using Total-seq A Hash Tag Oligonucleotide HTO antibodies (BioLegend; TotalSeq-A0251, 394601; TotalSeq-A0252, 394603; TotalSeq-A0253, 394605; and TotalSeq-A0254, 394607) according to the manufacturer’s instructions. After a 30-minute incubation at 4 ^°^C, cells were washed twice with resuspension buffer, counted, and pooled to a total of 40,000 cells (10,000 per condition), aiming for a final yield of 5,000 viable cells per sample after excluding doublets and apoptotic cells in downstream analysis (https://satijalab.org/costpercell/). The pooled samples were loaded onto one lane of Chromium Chip B (10x Genomics, PN-1000074). HTO and cell libraries were prepared following Chromium Single Cell 3’ Kit v3.1 (10x Genomics, PN-1000484) protocols and sequenced on Illumina NextSeq 500 platform using the NextSeq 500/550 High Output Kit v2.5 (75 cycles; Illumina, cat. 20024906) with a total coverage of ~609 M reads.

### Analysis of scRNA-seq data

The Cell Ranger Single-Cell software Suite (https://support.10xgenomics.com/single-cell-gene-expression/software/pipelines/latest/what-is-cell-ranger) was used to perform alignment to the genome (GRCh38), barcode assignment for each cell, gene counting by unique molecular identifier (UMI), and sample demultiplexing based on the TotalSeq A hashing indexes (hash index AGTAAGTTCAGCGTA (TotalSeq-A0254) for naive PBMCs (PBS), hash index GTCAACTCTTTAGCG (TotalSeq-A0251) for *S*.Tm D23580 infected PBMCs, hash index TGATGGCCTATTGGG (TotalSeq-A0252) for *S*.Ty infected PBMCs, and hash index TTCCGCCTCTCTTTG (TotalSeq-A0253) for *S*.Pty A infected PBMCs). A total of 24,819 cells captured in GEM droplets were retrieved, with a mean of 22,839 reads per cell and a median of 1,137 genes per cell. Doublets containing more than one unique HTO tag were removed, and cells with a high percentage of mitochondrial genes (more than 20%) were excluded. Overall, 12,907 cells were retained for downstream analysis, including 3155 naïve cells, 3261 *S*.Tm-infected cells, 3015 *S*.Ty-infected cells, and 3476 *S*.Pty-infected cells; all cells had a total count of at least 250 UMIs.

Naïve and infected cells were analyzed separately. For each dataset, data were normalized to a library-size factor, calculated as the median of the total UMI counts across all cells divided by the total UMI counts in each cell. Normalized data were log2-transformed. Cell cycle and ribosomal genes were filtered out before selecting variable genes. Variable genes were selected based on fitting the data to a simple noise model based on the gene’s mean expression and dispersion (coefficient of variance) across all single cells. Principal component analysis (PCA) was performed on the variable genes, and the first 13 PCs for naïve cells and 15 PCs for infected cells were selected for downstream analysis based on the inflection point of the curve of the variance explained by each successive principal component. tSNE in the PC space was calculated to visualize the data. The Euclidian distance in the PC space and the Jaccard similarity were used to build a k-nearest neighbor (KNN)-graph with k = 30. Unsupervised clustering of the single cells was done using Louvain community detection. We used an iterative implementation of the Louvain method to optimize the modularity-like quality function by iterating the Louvain algorithm until convergence. Cluster identity was inferred using cluster-specific genes and cell-type marker genes. Next, we re-cluster each cell type to the subset level using the Louvain algorithm and obtained a total of 12 subsets in the naïve cells and 21 subsets in the infected cells.

### Bacterial growth

All strains were grown in LB medium without antibiotics unless otherwise specified. For experiments with typhoidal strains (Typhi, Paratyphi A), the bacterial strains were grown for 14 h at 37 ^°^C with rotation in 15 mL conical tubes (Eppendorf 0030122160) for extra safety precautions, to early stationary phase. In experiments conducted with non-typhoidal strains, *Salmonella* cultures were incubated for 16h at 37°C in snap-cap 14 ml tubes (Falcon, 352051) in an aerobic position. For log-phase cultures, bacteria from overnight starters were diluted 1:100 into fresh media and grown for 2h at 37°C with rotation.

For growth conditions used in ELISPOT assays and DMRM-MS, see the corresponding sections in the Methods.

### Growth curves

M9 minimal medium (6 g/L Na_2_HPO_4_, 3 g/L KH_2_PO_4_, 0.5 g/L NaCl, 1 mL/L CaCl_2_ 0.1M, 1 mL/L MgSO_4_ 1M, and 5mg/L thiamine) was supplemented to final concentrations of 0.4% glucose, 50 µg/mL kanamycin, and 1 mM IPTG (hereafter referred to as M9+). Experiments involving riboflavin (RF) were performed using freshly prepared RF solutions and protected from light. Riboflavin (Merck R4500) was dissolved in 0.1 N NaOH to generate a 10 mg/mL stock solution. This stock was subsequently diluted in M9 + medium to final concentrations of 100 µg/mL, 20 µg/mL, 10 µg/mL, or without RF.

CRISPRi-dCas9 hypomorph strains were grown overnight in LB medium supplemented with 50 µg/mL kanamycin. Cultures were then washed twice with PBS+ to remove residual riboflavin from the LB medium and diluted to an OD_600_ of 0.1 in PBS + . The bacterial cultures were subsequently added to M9 + medium containing the indicated RF concentrations at a 1:100 dilution from the OD_600_ = 0.1 cultures. Each condition had 6 replicates; growth was measured by OD_600_ reads in a 96-well plate at 24-minute intervals for 39 hours in FLUOstar Omega plate reader (BMG). Lag-time was calculated by the linear tangent method on ln(OD) (Zwietering method).

### Collection of supernatants

To evaluate MAIT cell responses to bacterial supernatants, bacterial cultures were grown overnight in 30 mL LB at 37 °C with shaking (160 rpm). The following day, cultures were adjusted to the same OD_600_ as the sample with the lowest measured density, assuming that bacterial density correlates with the concentration of secreted ligands in the medium.

Cultures were transferred to 50 mL tubes and centrifuged at 3,000 × g for 10 minutes to pellet bacteria. Supernatants were carefully transferred to fresh tubes, and this centrifugation step was repeated three times to ensure complete removal of bacterial cells. The resulting supernatants were then filtered through 0.22 μm Millex-GV syringe filter units (Sigma-Aldrich, SLGV033RS). To confirm sterility, 100 μL of each filtered supernatant was plated on LB agar plates without antibiotics and incubated overnight at 37 °C.

For MAIT cell stimulation, supernatants derived from *S*.Tm strains 4/74 and D23580 were added to PBMC cultures at volumes of 30, 50, or 70 μL in a total of 200 μL. For Rib^KD^ hypomorph-derived supernatants, 30 μL was added to PBMC cultures in total of 130 μL. For ELISPOT assays, see the ‘ELISPOT assays’ section in the Materials and Methods.

### Flow cytometry analysis

PBMCs were thawed and rested overnight (see “PBMC extraction and treatment” in Materials and Methods). Four hours post-infection, cells were treated with 1 µg/mL Brefeldin A (GolgiPlug BD Biosciences, 51–2301KZ) to inhibit protein secretion and allow intracellular accumulation of IFNγ and TNFα. Cells were incubated at 37 °C with 5% CO_2_ for up to 17 hours. The following day, cells were pelleted by centrifugation at 500 × g for 5 minutes, and the media was carefully aspirated. Cells were washed twice with PBS-/-. After the second wash, cells were resuspended in 5 mM EDTA in PBS-/- and incubated for 10 minutes to promote detachment. Cells were then centrifuged (500 × g, 5 minutes), the supernatant discarded, and resuspended in FACS buffer (PBS-/- supplemented with 10 mM EDTA and 5% heat-inactivated FBS). To prevent non-specific antibody binding, Human Fc Block (BD Biosciences, 564220) was added, and cells were incubated for 10 minutes at 4 °C. Cells were then centrifuged (500 × g, 5 minutes, 4 °C). Viability staining was performed using Zombie Aqua (BioLegend, 423102) or FVS450 (BD Biosciences, 562247), followed by surface staining with antibodies against CD3-FITC (clone UCHT1, BioLegend, 300406) and CD161-PerCP-Cy5.5 (clone HP-3G10, BioLegend, 339908). Cells were incubated for 20–30 minutes at 4 °C in the dark.

Following surface staining, cells were washed twice with PBS-/- and fixed and permeabilized using the Cytofix/Cytoperm kit (BD Biosciences, 555028) according to the manufacturer’s instructions. Intracellular staining was then performed using antibodies against Vα7.2-BV605 (clone 3C10, BioLegend, 351720), TNFα-PE (clone Mab11, BioLegend, 502909), and IFNγ-PE-Cy7 (clone S.B3, BioLegend, 502528). Cells were incubated for 30 minutes at 4 °C in the dark. Finally, cells were washed three times, resuspended in 200 µL FACS buffer, and analyzed by flow cytometry using a FACSAria III cytometer with DIVA software (BD Biosciences). Data were analyzed using FlowJo software (v10.7.2). Antibodies were used at 1 µL per 5 × 10^5^ cells in 100 µL staining volume, except for CD161 (2 µL) and Vα7.2 (2.5 µL).

### RF biosynthesis pathway in *Salmonella*

The production pathway (Fig 4A) was summarized using public data sources, with focus on BioCyc (Pathway ID RIBOSYN2-PWY) and KEGG (Pathway IDs STM474_1726 and STMMW_17061).

### Generation of CRISPRi hypomorphs

CRISPRi-dCas9 inducible gene knockdown was performed based on a previously published protocol [43]. Single guide RNA (sgRNA) sequences were designed using CHOPCHOP (https://chopchop.cbu.uib.no/) and verified to match the corresponding regions in *Salmonella enterica* serovar Typhimurium strains 4/74 and D23580. A non-targeting (scramble) sgRNA with no complementarity to the *Salmonella* genome was used as a control (Addgene Plasmid #1864). Oligonucleotides (see S2 Table) encoding sgRNA sequences were synthesized (Sigma) with appropriate overhangs for BsaI-digested plasmid pJMP1339 (Addgene Plasmid #11927), which carries ampicillin and kanamycin resistance markers. Oligonucleotides for sgRNA were phosphorylated using T4 polynucleotide kinase (NEB, M0201S) and ligated into the vector using T4 DNA ligase (NEB, M0202L). The ligation product was transformed into diaminopimelic acid (DAP; Sigma, 583-93-7)-dependent E. coli MFDpir by electroporation. Transformants were recovered in SOC medium supplemented with 0.3 mM DAP and plated on LB agar containing 100 μg/mL ampicillin and 0.3 mM DAP. Colonies were screened by colony PCR to confirm sgRNA insertion, and positive clones were grown in LB supplemented with ampicillin, kanamycin, and DAP. For conjugation, MFDpir carrying pJMP1339-sgRNA and MFDpir carrying the dCas9-expressing plasmid pJMP1039 (Addgene plasmid #119239) were mixed with recipient *Salmonella* strains (4/74 or D23580) at a 1:1:1 ratio. The mixture was plated on LB agar containing 50 μg/mL kanamycin without DAP to counter-select donor strains and select for successful transconjugants. Plates were incubated overnight at 37°C. Colonies were collected, and the presence of sgRNA constructs was confirmed by Sanger sequencing. See the used primers in S2 Table.

### qRT-PCR

Hypomorph strains were grown overnight in LB medium supplemented with 50 μg/mL kanamycin. Cultures were diluted 1:100 into fresh LB containing 50 μg/mL kanamycin and grown for approximately 3.5 hours to mid-log phase (OD_600_ = 0.3–0.6), in the presence or absence of 1 mM IPTG. Three biological replicates were prepared per condition. Bacterial cultures were pelleted and lysed in RLT buffer (QIAGEN Buffer RLT, 79216), and samples were stored at -80°C until RNA extraction. Total RNA was purified using the RNeasy Mini Kit (Qiagen, 74104), including on-column DNase treatment (Qiagen RNase-Free DNase, 79254) to remove genomic DNA contamination. cDNA was synthesized using qScript cDNA SuperMix (Quantabio, 95048-100) according to the manufacturer’s instructions. Quantitative PCR (qPCR) was performed using Fast SYBR Green Master Mix (ThermoFisher Scientific, 4385616). Gene expression levels of target *rib* genes were normalized to the housekeeping gene *gapA.*

Relative expression was calculated using ΔΔCt method, where ΔCt = Ct(target gene) − Ct(reference gene *gapA*), and ΔΔCt = ΔCt(sample) – average(ΔCt(IPTG non-induced (-IPTG) condition in the three biological replicates)). Fold change in gene expression was calculated as 2^(-ΔΔCt)^. Each sample was analyzed with three technical qPCR replicates. Results are presented as the mean ± standard error of the mean (SEM) of fold change values comparing IPTG-induced (+IPTG) and non-induced (-IPTG) conditions to the average of -IPTG.

### Construction of RibH expressing plasmid

A tetracycline-resistant expression vector (pTcR) was constructed to enable constitutive gene expression under TetR. The vector backbone was assembled by Gibson Assembly x2 Master Mix (NEB, E2611L) by amplification of the origin of replication (ORI) from pBAD24 (Beckwith Lab [67]), a constitutive *rpsM* promoter from pFPV25 (Addgene Plasmid #20667), and a tetracycline resistance cassette from pRed/ET (GeneBridges, A104). The construct pTcR was designed to contain a single EcoRI restriction site downstream of the *rpsM* promoter and TetR to facilitate insertion of target genes. TetR was amplified with an additional EcoRI cut site and an additional 8 nt on either side.

The plasmid was assembled using Gibson Assembly and transformed into competent *E. coli* DH5α by heat shock. Transformants were selected on LB agar supplemented with 10 μg/mL tetracycline, and plasmid integrity was confirmed by Sanger sequencing.

For RibH overexpression, a native RibH sequence was edited to contain a silent mutation every 3^rd^ codon at the dCas9-CRISPRi binding site, to prevent IPTG-induce silencing of plasmid-derived RibH. Specifically, the original sgRNA recognition sequence (CTGACGCTCGTGTCGCCATC) was replaced with a synonymous sequence (C**G**GA**T**GC**G**CG**C**G**TTGCA**A**TT**), thereby disrupting sgRNA binding while preserving protein sequence. Synthetic gene fragments (Twist Bioscience) encoding the modified sgRNA target site were cloned into EcoRI-digested pTcR using Gibson Assembly. Constructs were verified by Sanger sequencing and introduced into *Salmonella* RibH knockdown hypomorph strains by electroporation. Transformants were selected on LB agar plates containing 10 μg/mL tetracycline and 50 μg/mL kanamycin. See primers in S2 Table.

To improve the yield of pTcR, an additional HindIII cut site (AAGCTT) and 4 random nt on both sides was constructed into pTcR by Gibson Assembly (See S2 Table).

To express the transporter RibN from *Rhizobium leguminosarum* (*RL1692*), a synthetic gene fragment (Twist Bioscience) was amplified with handles for Gibson Assembly within the new pTcR (hereafter npTcR) and expressed in competent *E. coli* DH5α. Following extraction and Sanger validation, npTcR-RibN plasmid was transformed and expressed within *S*.Tm D23580 strain.

### Intracellular survival assay

To assess intracellular survival of *Salmonella* RibH^KD^ and RibE^KD^ hypomorphs in PBMCs, colony-forming units (CFU) were quantified at 4 and 24 hours post-infection. At the indicated time points, infected PBMCs were washed twice with PBS- and incubated for 10 minutes in buffer containing 5 mM EDTA in PBS-/-. Following this, 0.1% Triton-100x was added at 1:1 ratio and mixed vigorously by pipetting to lyse host cells and extract intracellular bacteria. Lysates were incubated for 10 minutes to ensure complete release of intracellular bacteria. Samples were serially diluted (1:5) into PBS-/- and plated onto LB agar supplemented with 50 μg/mL kanamycin. Plates were incubated overnight at 37°C, and colonies were counted the following day; CFU/ml were calculated based on colony counts, adjusted for the dilution factor, the fraction of the sample plated, and the total volume of the lysate.

### Protein detection and analysis

Bacterial cultures were grown in LB + 50 ug/mL kanamycin with and without IPTG overnight in three replicates, then diluted to a density of approximately 10^9^ cells/mL, and pellets from 1 ml were collected, snap-frozen in liquid nitrogen, and stored at -80°C until further processing. Samples were subjected to in-solution tryptic digestion using the S-Trap protocol [68]. The resulting peptides were analyzed by nanoflow liquid chromatography (NanoAcquity, Waters) coupled to high-resolution, high-mass-accuracy mass spectrometry (Thermo Exploris 480, Thermo Fisher Scientific). Each sample was analyzed individually in randomized order using data-dependent acquisition (DDA) mode. Raw data were processed using the MetaMorpheus (v1.0.2) informatics platform. Spectra were searched against the *Salmonella enterica* serovar Typhimurium strain 4/74 proteome database, downloaded from UniProt, and supplemented with common laboratory contaminant proteins. Rib proteins were included in the reference database, and no additional custom sequences were required.

### MR1T and BEAS-2B cells

BEAS-2B cells were obtained from ATCC. BEAS-2B MR1 KO cells were generated previously using CRISPR/Cas9 and have been described before [69]. Both BEAS-2B cell lines were cultured in Dulbecco’s Modified Eagle Medium (Gibco) supplemented with 10% heat-inactivated fetal bovine serum (GeminiBio) and 2% L-Glutamine (Gibco). The TRAV1-2 + MAIT cell clone D426-G11 was derived from an individual latently infected with Mtb and has been extensively characterized [70,71]. It was expanded and maintained as before and used from frozen.

To assess infection efficiency of the hypomorphs in BEAS-2B cells, infections were performed similarly to the ELISPOT experiments. In short, Salmonella strains were recovered from glycerol stocks into LB broth (Invitrogen, 12795-027) and incubated for 5–6 h at 37°C, shaking, before induction with 1 mM IPTG (Sigma, I6758-5G) overnight. Bacterial cultures were brought to the same OD600 and centrifuged at 17,000 x g for 10 min, washed in DPBS (Fisher Scientific, 14-190-144) and brought to the same OD600 again. 2 x 10^5^ BEAS-2B cells per well were plated in DMEM supplemented as above in 6-well plates and infected with the different Salmonella strains at an MOI of 20 based on a conversion of OD600 = 1 equals 10^9^ CFU/ml. After 2 hours of incubation at 37°C, gentamycin (Gibco, 15750078) was added to a final concentration of 50 μg/ml. The final concentration of IPTG in the wells was 1 mM. On the next day, equal cell numbers were lysed in water and lysates were diluted in DPBS before plating on LB agar plates (Fisher Scientific, BP1425-500) containing Kanamycin (USBiological Life Sciences, 17924; for hypomorphs) or not (for WT controls). Colonies were counted on the following day and CFU/1 x 10^5^ cells were calculated.

### ELISPOT assays

96-well mixed cellulose esters enzyme-linked immunosorbent spot (ELISPOT) plates (Millipore, MSHAS4510) were coated with IFN-γ capture antibody clone 1-D1K (Mabtech, 3420-3-1000) in 0.1M Na2CO3 (Sigma-Aldrich, S-2127), 0.1M NaHCO3 (Sigma-Aldrich, S-8875), pH = 9.6 at 4°C overnight. Plates were washed three times with sterile phosphate buffered saline (PBS; Corning, 21-040-CV) and blocked with Roswell Park Memorial Institute (RPMI) 1640 (Gibco, 22400105) supplemented with 10% heat-inactivated human serum (prepared in house) and 2% L-Glutamine (Gibco, 25030164) for at least one hour at room temperature. Blocked plates were stored at 4°C for a maximum of one week. Salmonella strains were recovered from glycerol stocks into LB broth (Invitrogen, 12795-027) and allowed to recover for 5–6 h at 37°C, shaking. For hypomorph strains, 50 μg/ml Kanamycin (USBiological Life Sciences, 17924) was added to the LB broth. Cultures were subsequently diluted 1:1000 in fresh LB broth with or without Kanamycin and CRISPRi expression was induced with 1 mM of IPTG (Sigma-Aldrich, I1284-5ML) overnight. After 15-16h of incubation at 37°C with shaking, bacterial cultures were transferred to 4°C until ELISPOT plates were ready. For this, 1 x 10^4^ BEAS-2B cells and 50 μl of 4x concentrated IPTG or medium were added to each well of the ELISPOT plate such that the final concentration after addition of antigen and T cells was 1 mM during the overnight co-culture. Bacterial cultures were brought to the same OD600 and centrifuged at 17,000 x g for 10 min. Supernatants were centrifuged again before sterile-filtering through 0.22 μm PVDF centrifugal filter units (Millipore, UFC30GV0S). Bacterial pellets were resuspended in DPBS (Fisher Scientific, 14-190-144) and brought to the same OD600 before dilution in RPMI supplemented as above. Bacteria were added to the ELISPOT plate at an MOI of 20 based a conversion of OD600 = 1 equals 10^9^ CFU/ml, supernatants were at 50 μl/well, and the positive control PHA (Roche, 11249738001) was at 100 μg/ml. BEAS-2B cells were incubated with the respective antigens for 2 hours at 37°C before addition of 2.5 x 10^3^ T cells/well and gentamycin (Gibco, 15750078) to a final concentration of 50 μg/ml. Cells were co-cultured at 37°C for at least 18h. Plates were washed extensively in DPBS (Sigma Aldrich, D5652-50L) + 0.05% Tween-20 (Affymetrix, 20605) and then incubated with alkaline phosphatase-conjugated secondary antibody clone 7-B6-1-ALP (Mabtech, 3420-9A-1000) in PBS supplemented with 0.5% BSA (Fisher Scientific, BP1600-100) and 0.05% Tween-20 for at least two hours at room temperature. Plates were washed in PBS + 0.05% Tween-20 again and developed using BCIP/NBT-plus developer (Mabtech, 3650-10). IFN-γ spots were enumerated using an AID ELISPOT reader and AID EliSpot Software Version 7 (Autoimmun Diagnostika GMBH).

### Liquid chromatography -MS/MS analysis

Cells and supernatant were prepared for mass spectrometry as previously described [40]. Briefly, cells were resuspended in Acetonitrile/Methanol/Water (2:2:1) and transferred to screw-cap homogenization tube prefilled with lysing matrix B. Cells were disrupted using the TissueLyser LT homogenizer (QIAGEN, 85600) at 50 oscillations/sec for 60 seconds (10 rounds), cooling on ice for 2 minutes between cycles. Homogenized cells were centrifuges at 15000 x g for 20 minutes and supernatant collected and dried down in speed vacuum centrifuge and weight determined. Supernatants were diluted with equal volume of Acetonitrile/Methanol/Water (2:2:1) and dried down in speed vacuum centrifuge and weight determined. Dried samples were then resuspended in Acetonitrile/Methanol/Water (2:2:1) to make a final concentration of 20 mg/ml and 100 mg/ml for cell and supernatant respectively. Samples were analyzed using the Agilent 1290 Infinity III Liquid Chromatography System attached to a 6490 Triple Quadrupole MS system. 2 µl of samples were loaded per injection. Separation was performed on the InfinityLab Poroshell 120 EC-C18 Column (Agilent, 695975–906) (4.6 × 100 mm, 2.7 μm). 0.1% formic acid in water and 0.1% formic acid in acetonitrile were used as mobile phase A and B respectively. Separation was carried out using gradient elution of 2% solvent B held for 1.5 minutes and then 20% solvent B over a duration of 3.5 minutes, then 20% solvent B to 99% solvent B for 3 minutes. 99% solvent B was held for 0.5 minutes and then returned to 2% solvent B for 2.5 minutes for re-equilibration. Mass spectra and tandem mass spectra were carried out as previously described [40].

### Targeted metabolomics analysis by dynamic multiple reaction monitoring-mass spectrometry (DMRM-MS)

For MRM metabolomics Bacteria were grown as described in the ELISPOT section. After overnight induction, cultures were stored at 4°C until processing. 1 ml of each culture was centrifuged at 17,000 x g for 10 min. Supernatants were centrifuged again before sterile-filtering through 0.22 μm PVDF centrifugal filter units (Millipore, UFC30GV0S). Bacterial pellets and supernatants were stored at -80°C and shipped on dry ice.

DMRM was built for all compounds as previously described [40,72]. Briefly, standards for DMRL, riboflavin, FMN and FAD at 1 µg/ml were used to determine optimal fragmentor voltage and collision energy for each compound. Top three or four product ions based on intensity were selected for each compound and retention time determined. Samples were analyzed via DMRM using LC-MS/MS method described. Raw data for each injection was imported into Agilent Masshunter Qualitative Analysis Software version 10.0. Peaks for selected product ions were integrated for each compound and adjusted if necessary. Total peak area for most stable product ion was manually extracted and used for quantification. Details of precursor and transition ions used for DMRM are shown in S3 Table.

## Supporting information

S1 Fig scRNA sequencing uncovered serovar-specific MAIT activation. (**A**) Dot-plot of marker genes used to identify immune cell populations. Dot size represents the percentage of cells in a subset expressing a given gene, and dot color indicates relative expression levels (legend at the right) (**B**) Distribution of immune cell population across experimental conditions.
(EPS)

S2 Fig Flow cytometry analysis confirmed serovar- and lineage- specific MAIT activation. (**A**) Gating strategy for flow cytometry of activated MAIT (IFNγ^+^ or TNFɑ^+^) from total MAIT cells (CD3^+^CD161^+^Vɑ7.2^+^) of infected PBMCs. (**B + C**) Bar plots representing flow cytometry analysis of total MAIT from naïve PBMCs or PBMCs infected with ST19 (4/74), ST313 lineage 1 (L1; A130, A1339), lineage 2 (L2; D23580, Kinshasa), lineage 2.2 (L2.2; D36233, D37712), and lineage 3 (L3; BKQZM9, CAA8By).
(EPS)

S3 Fig Bacterial factors mediated strain-specific MAIT activation. (**A**) Flow cytometry analysis of total MAIT from naive PBMCs or PBMCs infected with log phase or stationary 4/74 and D23580. (**B)** Flow cytometry analysis of total MAIT from PBMCs treated with αMR1 blocking antibody or IgG isotype control, and either left uninfected or infected with 4/74 and D23580. (**C**) Flow cytometry analysis of total MAIT from naive PBMCs or PBMCs infected with live or PFA-fixed 4/74 and D23580 at MOI 0.1, 1, 10 or 50. (**D**) Flow cytometry analysis of total MAIT from PBMCs treated with bacterial supernatants from 4/74 and D23580 in concentrations of 15%, 25% or 35% of total culture volume.
(EPS)

S4 Fig Genetic perturbations of *Salmonella rib* enzymes revealed strain-specific growth inhibition. (**A**) Growth curves measurements of the indicated *rib* hypomorphs from 4/74 (blue) and D23580 (green). Bacteria were grown in M9 minimal media in the presence of IPTG and RF (0, 10, 20, 100 µg/ml). Optical density (OD_600_; Y-axis) were measured at 24-minute intervals over time (X-axis), with 6 replicates for each condition. (**B**) CFU measurements of RibH hypomorph from 4/74 (blue) and D23580 (green). Bacteria were grown in M9 minimal media in the absence or presence of IPTG and RF (0, 10, 20, 100 µg/ml). (**C**) Growth curves of D23580 RibB^KD^ carrying a plasmid encoding the RF transporter RibN. Cultures were grown in minimal media, without IPTG (in grey) or with IPTG and supplemented with RF (0, 10, 20, 100 µg/ml).
(EPS)

S5 Fig Strain-specific RibH^KD^ and RibE^KD^ effects on MAIT activation. (**A**) Flow Cytometry of activated MAIT cells from naïve PBMCs or PBMCs infected by 4/74 (blue) or D23580 (green), carrying an empty vector or a vector expressing RibN. (**B-D**) Proteomics analysis of RibH^KD^ in 4/74 (blue) or D23580 (green). Presented are PCA plot (**B**), volcano plot of differentially expressed proteins (**C**), and enriched pathways and regulons of differentially expressed proteins (**D**). Statistics: P-values were corrected for multiple testing with Benjamini–Hochberg FDR (5%FDR and 1.5fold for the volcano plots in (C), and 10%FDR for pathway enrichment in (D)).
(EPS)

S6 Fig RibH and its substrate 5-A-RU were the main source for differential strain-specific MAIT activation. (**A**) MR1T cells were co-incubated with BEAS-2B MR1^KO^ (upper panel) or without APCs (lower panel) as controls, and infected with strains D23580 and 4/74: WT, scramble, RibH^KD^ or RibE^KD^ hypomorphs. MAIT activation was measured by IFN-γ enzyme-linked immunospot (ELISPOT) assay. Phytohemagglutinin (PHA) was added as positive control. (**B**) BEAS-2B cells were infected with WT, scramble, RibH^KD^, or RibE^KD^ hypomorphs of strains D23580 or 4/74 overnight. Cells were lysed and diluted lysates were plated to allow CFU counts. Data are pooled from three (two for the D23580 WT) independent experiments for each of the two sets of *Salmonella* strains. (**C**) Levels of RF, FMN, and FAD from RibE^KD^ and RibH^KD^ in 4/74 and D23580 was quantified using MRM. Quantification was performed using chromatographic peak area. (**D**) RNA was isolated from cultures of RibH^KD^ in 4/74 and D23580, carrying a plasmid expressing RibH (pRibH) or an empty vector. Presented are qRT-PCR analyses using primers to the *RibH* gene, presented as average fold change between +IPTG/-IPTG (Y-axis). (**E**) Flow cytometry analysis of total MAIT from naïve PBMCs or PBMCs infected with RibH^KD^ 4/74 (blue) or D23580 (green) carrying a plasmid expressing RibH (pRibH) or an empty vector (cont.). (**F-G**) PBMCs were infected with RibH^KD^ (**F**) or RibE^KD^ (**G**) 4/74 and D23580, and at 4 or 24 hours post infection cells were lysed and intracellular bacteria were quantified by plating on agar plates. (**H**) Flow cytometry analysis of total MAIT from naïve PBMCs or PBMCs infected with RibH^KD^ in 4/74, D23580, D36233, D37712, BKQZM9 and CAA8BY. Statistics: paired t-test between with and without IPTG in (A-C), Student t-test for (D-H) (*) *p* < 0.05; (**) *p* < 0.001; (***) *p* < 0.0005.
(EPS)

S1 Table Protein abundance with and without IPTG in RibH-KD strains. (XLSX)

S2 Table List of primers used in this study. (XLSX)

S3 Table Multiple Reaction Monitoring (MRM) results for RF intermediates. (XLSX)
